# Delivery of pleasant stroke touch via robot in older adults

**DOI:** 10.3389/fpsyg.2023.1292178

**Published:** 2024-01-09

**Authors:** Tomoki Ishikura, Wataru Sato, Jun Takamatsu, Akishige Yuguchi, Sung-Gwi Cho, Ming Ding, Sakiko Yoshikawa, Tsukasa Ogasawara

**Affiliations:** ^1^Robotics Laboratory, Division of Information Science, Nara Institute of Science and Technology, Ikoma, Japan; ^2^Psychological Process Research Team, Guardian Robot Project, RIKEN, Kyoto, Japan; ^3^Applied Robotics Research, Microsoft Corporation, Redmond, WA, United States; ^4^Robotics Laboratory, Department of Medical and Robotic Engineering Design, Faculty of Advanced Engineering, Tokyo University of Science, Tokyo, Japan; ^5^Knowledge Acquisition and Dialogue Research Team, Guardian Robot Project, RIKEN, Kyoto, Japan; ^6^Division of Electronic Engineering, School of Science and Engineering, Tokyo Denki University, Saitama, Japan; ^7^Institutes of Innovation for Future Society, Nagoya University, Nagoya, Japan; ^8^Institute for Philosophy and Science of Art, Kyoto University of the Arts, Kyoto, Japan

**Keywords:** robot touch, older adults, subjective and physiological response, facial electromyogram (EMG), valence and arousal ratings

## Abstract

Touch care has clinically positive effects on older adults. Touch can be delivered using robots, addressing the lack of caregivers. A recent study of younger participants showed that stroke touch delivered via robot produced subjective and physiologically positive emotional responses similar to those evoked by human touch. However, whether robotic touch can elicit similar responses in older adults remains unknown. We investigated this topic by assessing subjective rating (valence and arousal) and physiological signals [corrugator and zygomatic electromyography (EMG) and skin conductance response (SCR)] to gentle stroking motions delivered to the backs of older participants by robot and human agents at two different speeds: 2.6 and 8.5 cm/s. Following the recent study, the participants were informed that only the robot strokes them. We compared the difference between the younger (their data from the previous study) and the older participants in their responses when the two agents (a robot and a human) stroked them. Subjectively, data from both younger and older participants showed that 8.5 cm/s stroking was more positive and arousing than 2.6 cm/s stroking for both human and robot agents. Physiologically, data from both younger and older participants showed that 8.5 cm/s stroking induced weaker corrugator EMG activity and stronger SCR activity than the 2.6 cm/s stroking for both agents. These results demonstrate that the overall patterns of the older groups responses were similar to those of the younger group, and suggest that robot-delivered stroke touch can elicit pleasant emotional responses in older adults.

## 1 Introduction

Given the increasing proportion of older adults in societies worldwide, the lack of caregivers is becoming an urgent issue. Additionally, the importance of skillful or therapeutic touch in medical applications is receiving increasing attention (Arnould-Taylor, 1991; Gineste and Pellissier, 2007; Field, 2019; Jagan et al., 2019; Grandi and Bruni, 2023). Several observational studies have found that touching patients during e.g., massage produces several positive effects, including a reduction in pain (Goldstein et al., 2016) and an improvement in sleep (Andersson et al., 2009). In older adults, *Humanitude* care, including touching, has reportedly reduced the frequency of neuropsychiatric symptoms associated with dementia compared with conventional care (Honda et al., 2016).

Several studies have shown that touching interactive robots provides emotional positive effects, such as reductions in pain and stress (Geva et al., 2020, 2022). Besides, if such forms of the aforementioned skillful touch could be delivered by a robot, care receivers could have emotional positive effects and the lack of caregivers might be resolved (Eckstein et al., 2020; Sumioka et al., 2021). A recent study (Ishikura et al., 2023) measured the subjective ratings (valence and arousal) and physiological signals [facial electromyography (EMG) recorded from the corrugator supercilii and zygomatic major muscles related to brow lowering and lip corner pulling actions, respectively, and skin conductance response (SCR)] of younger participants to 20 s of gentle stroking delivered by a robot compared with that delivered by a human at two different speeds (2.6 vs. 8.5 cm/s). Several previous psychophysiological studies have revealed that the facial EMG activities of these muscles and electrodermal activity can provide physiological signals associated with subjective valence and arousal, respectively (Greenwald et al. 1989; Lang et al. 1993; Sato et al. 2020; Sato and Kochiyama 2022; for reviews, see Cacioppo et al. 1992; Lang et al. 1998). Studies that investigated the effect of touch using brushes also showed that the facial EMG activities of these muscles (Pawling et al., 2017; Mayo et al., 2018; Ree et al., 2019) and SCR (Ree et al., 2019) indicated pleasant effects of touch. These speeds were expected to activate the C tactile (CT) system (1.0–10.0 cm/s), although the optimal speed can differ across different body regions (Ackerley et al., 2014). For both human and robot agents, the 8.5 cm/s stroking elicited more positive subjective (i.e., higher valence ratings) and physiological (i.e., reduced corrugator supercilii EMG) responses compared with the 2.6 cm/s stroking (Ishikura et al., 2023). The similarities in the speed-dependent modulations of touch across the agents imply that robots can provide a pleasant stroke touch like humans.

However, whether robotic touch can elicit similar responses in older adults remains unknown. To our knowledge, no studies have tested the emotional effect of robotic touch, i.e., the subjective ratings and physiological signals by a robotic touch in older adults. On the other hand, several clinical studies reported that skillful or therapeutic touch delivered by a human can have positive effects on older adults. It was also proposed that the peripheral unmyelinated CT afferents are resistant to age-related degeneration (McIntyre et al., 2021). Thus, we hypothesized that a robot could deliver a pleasant touch to older and younger people.

In this paper, we tested the hypothesis by assessing subjective ratings (valence and arousal) and physiological signals (corrugator and zygomatic EMG and SCR) to gentle back stroking delivered to younger and older participants by a robot or a human.

## 2 Method

### 2.1 Participants

We recruited 30 healthy older Japanese men (mean ± SD age, 72.0 ± 4.2 years) from a human resource center in Kyoto, Japan. As in the previous study (Ishikura et al., 2023), we recruited only male participants because variable touch effects have been reported across genders (Russo et al., 2020). We jointly analyzed data collected from 28 younger Japanese men (mean ± SD age, 23.1 ± 2.9 years) in the previous study (Ishikura et al., 2023) in which an identical experiment was conducted. We calculated the demanded sample size using G*Power software ver. 3.1.9.2 (Faul et al., 2007). We assumed a two-way repeated-measures analysis of variance (ANOVA) with group as the between-participant factor and speed and agent as within-participant factors. The α level was 0.05, the power (β−1) was 0.80, the effect size (*f*) was 0.15 (small), and the correlation was 0.6. We found that more than 50 participants were needed in total and tested 58 (30 older + 28 younger) participants. The younger data comes from the previous study (Ishikura et al., 2023). We obtained written informed consent from all participants for participation in this study as approved by the ethics committee of the Nara Institute of Science and Technology, Ikoma, Japan, and as consistent with the Declaration of Helsinki.

### 2.2 Apparatus and stimuli

The apparatus, stimuli, and procedure were identical to those in the previous study (Ishikura et al., 2023). To provide a robotic touch, we used a UR3e collaborative robot arm produced by Universal Robots.^1^ We chose a robot with a small payload (3 kg) to ensure participant safety; this robot was specifically designed for human-robot collaborative work. In addition, we used a human-like robotic hand, which was developed by our group (Ishikura et al., 2023) to satisfy the following conditions: flexibility enough to fit naturally to the touched surface; stiffness and softness like a human hand; and warmness like a human hand. The robotic hand consisted of joints and heaters, bones, and mimetic body tissue material. A type of soft silicon (*HITOHADA GEL*, EXSEAL CO., Ltd.^2^) was used for the material and the temperature of the surface of the robotic hand was maintained at 35°C.

We attached the robotic hand to the robot arm and provided a physical stroke to each participant's back. We kept constant pressure to generate stroke motions. We asked all participants to wear the same patient wear as in the previous study over their inner wear to control stimuli to the back.

**Figure 1:**
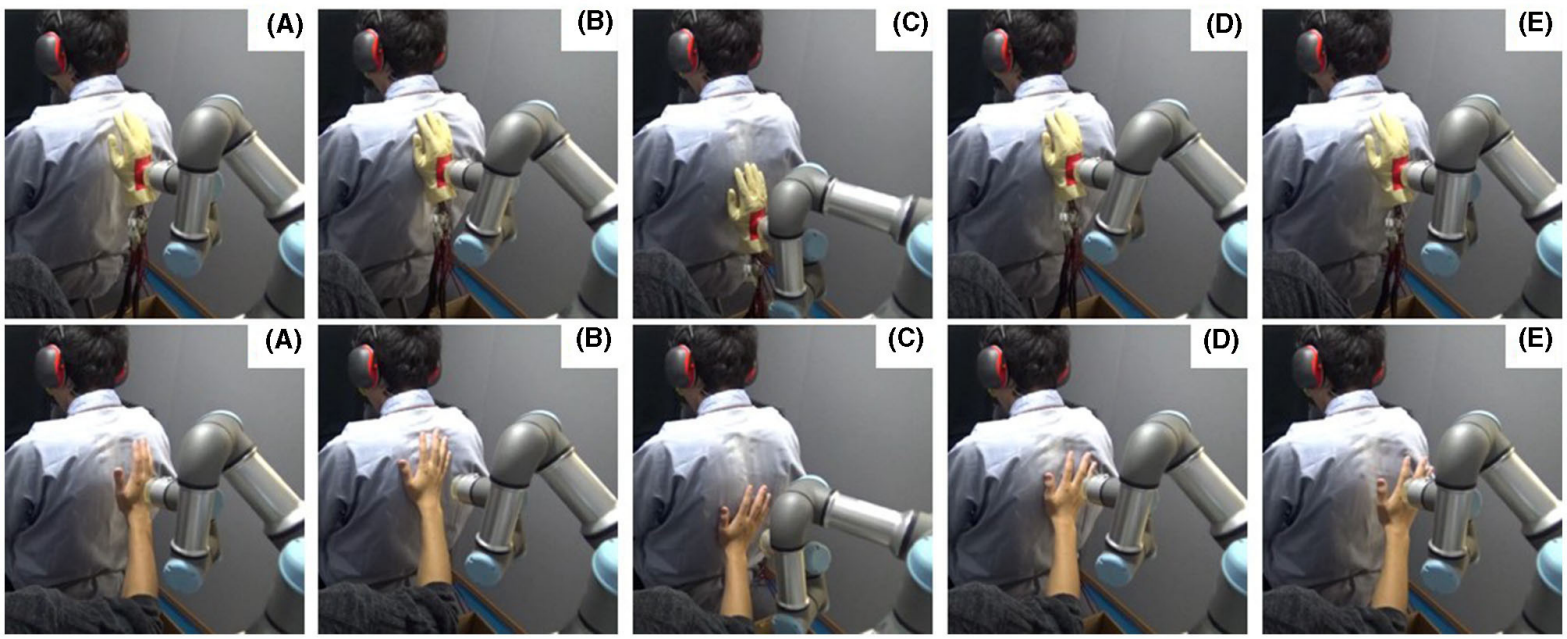
Experimental scene. **(A)** The agent moves toward the participant's back, **(B)** contacts the back and keeps pressure, **(C, D)** maintains a constant pressure while stroking the back with up and down, **(E)** and finally moves away from the back.

Figure 1 upper panels show the stroking by a robot. To stroke the back of a participant, the robot arm first moved toward the participant's back at 1 cm/s until it made contact. Then, it pressed the middle back of the participant. To give time for the participant to physiologically respond to the initial point of contact, the robot arm kept pressing in the same spot for 10 s. Next, it moved down the participants back by 15 cm and returned to the initial position. The robot arm repeated this motion for 20 s. After that, it moved away from the participant at 30 cm/s.

When stroking the participants back, the robot arm accelerated the robotic hand at 0.2 cm/s^2^ until it reached the predefined maximum speed, which was maintained for the rest of the motion. When approaching the goal position, the robot arm decelerated the robotic hand at 0.2 cm/s^2^. The average speed was set at 2.6 or 8.5 cm/s.

Figure 1 lower panels show the stroking motion performed by a human agent. When the human agent stroked the participant's back, the stroking speed was guided by the robot showing the movement to the human agent. Because it was difficult to convey the robot's tactile sensation in stroking motion to the human agent, the human agent practiced becoming familiar and consistent with the application of 3 N of force (the target force of the robot stroke) by training with a force plate before the experiment.

### 2.3 Procedure

The experimental design included the within-participants factors of touch agent (human and robot) and stroke speed [faster (8.5 cm/s) and slower (2.8 cm/s)]. We did not instruct the participants that there were two agents, rather instructed that only the robot stroked them every time, to test participants' pure evaluation of the stroke stimuli. Specifically, we wanted to prevent the participants from perceiving the existence of the human agent and thus evaluating the strokes according to whether a human or robot agent delivered them. The participant was unable to see the human or robot while the touch stimulus was being delivered to their back. To eliminate auditory cues, we programmed the robot to move even when the human was delivering the touch stimulus. Thus, the sound of the robot was kept constant across all conditions. Additionally, the participant wore earmuffs to decrease the amount of auditory information they received. After the experiment, we revealed whether a robot or human agent was performing the stroking motions on the back in the trials, and asked the participants whether they were aware of the existence of the human agent. None of the participants reported being aware of a human agent.

The participant was tested individually in a soundproofed room. First, to familiarize the participant with the robotic touch stimulus, we delivered the stimulus without ratings. Then, a total of 40 trials were conducted, with an equal number of agent and speed conditions. The order of conditions was pseudo-randomized. In each trial, the human/robot agent contacted the back of the participant and held the initial position for 10 s. Then, the stroke was delivered for 20 s at the speed of 2.6 or 8.5 cm/s. Finally, the participant subjectively rated the stimulus. Physiological signals were continuously measured during the experiment. After the 40 trials, the participant answered whether he/she was aware of the existence of the human agent in a free-description questionnaire.

Subjective valence and arousal ratings, representing qualitative and energetic components of subjective emotions, respectively, were obtained using the 9-point Affect Grid. For physiological evaluations, we recorded corrugator and zygomatic EMG and SCR. Corrugator and zygomatic EMG reflect emotional facial actions of brow lowering and lip corner pulling, respectively (Dimberg, 1990). In previous studies, subjective valence ratings showed linear negative and positive associations with cEMG and zEMG activity, respectively (Greenwald et al., 1989; Lang et al., 1993; Bradley and Lang, 2000; Larsen et al., 2003; Tan et al., 2012; Sato et al., 2013, 2020; Sato and Kochiyama, 2022). SCR reflects eccrine sweat gland activity, which is controlled by the sympathetic branch of the autonomic nervous system (Boucsein, 2011). Previous studies revealed a positive relationship between arousal ratings and SCR activity (Greenwald et al., 1989; Lang et al., 1993; Sánchez-Navarro et al., 2005; Neiss et al., 2009; Vico et al., 2010; van der Zwaag et al., 2011). For EMG recording, pairs of Ag/AgCl electrodes (diameter, 0.7 cm; interelectrode distance, 1.5 cm; Prokidai, Sagara, Japan) were placed on the participants left faces according to the guidelines (Fridlund and Cacioppo, 1986). The EMG signals were amplified and filtered (band pass: 20–400 Hz) using an EMG-025 amplifier (Harada Electronic Industry, Sapporo, Japan). For SCR recording, the Ag/AgCl electrodes (diameter, 1.0 cm; Nihonkoden, Tokyo, Japan) were attached to the index and middle fingers. The SCR signals were measured with a Model 2701 BioDerm Skin Conductance Meter (UFI, Morro Bay, CA, USA) while applying a constant voltage of 0.5 V between the fingers. The EMG and SCR signals were sampled at 1,000 Hz by using the LabChart Pro v8.0 software and PowerLab 16/35 data acquisition system (ADInstruments, Dunedin, New Zealand).

In addition, we assessed the attitudes toward robots using the Negative Attitude toward Robots Scale (NARS) and the Robot Anxiety Scale (RAS) (Nomura et al., 2008). The NARS assesses participants' attitudes toward robot behavior styles in human-robot interactions; higher scores denote more negative attitudes. The RAS can be used to determine whether anxiety is sufficient to prevent people from interacting with robots in daily life; higher scores indicate greater anxiety.^3^ We used these indices to determine whether negative attitudes toward or anxiety about robots affected participants' responses to being stroked by the robot.

### 2.4 Data analysis

Data preprocessing for physiological signals was conducted in the same way as in the previous study (Ishikura et al., 2023) using Psychophysiological Analysis Software 3.3 (Computational Neuroscience Laboratory of the Salk Institute, CA, USA) implemented in MATLAB 2021 (MathWorks, Natick, MA, USA). EMG data in each trial were sampled during the 10-s pre-stimulus and 20-s stimulus periods, re-rectified, baseline-corrected and averaged across the stimulus period. Then, the data ware standardized within individuals. The SCR data were preprocessed in the same manner without rectification.

Statistical analyses were conducted using JASP 0.14.1 (JASP Team, 2020). To assess our hypothesis, we applied repeated-measures ANOVAs with group (younger and older) as a between-participant factor and agent (human and robot) and speed [faster (8.5 cm/s)] and slower (2.8 cm/s) as within-participant factors for the subjective ratings and physiological signals. The results were regarded as statistically significant when *p* < 0.05.

## 3 Results

**Figure 2:**
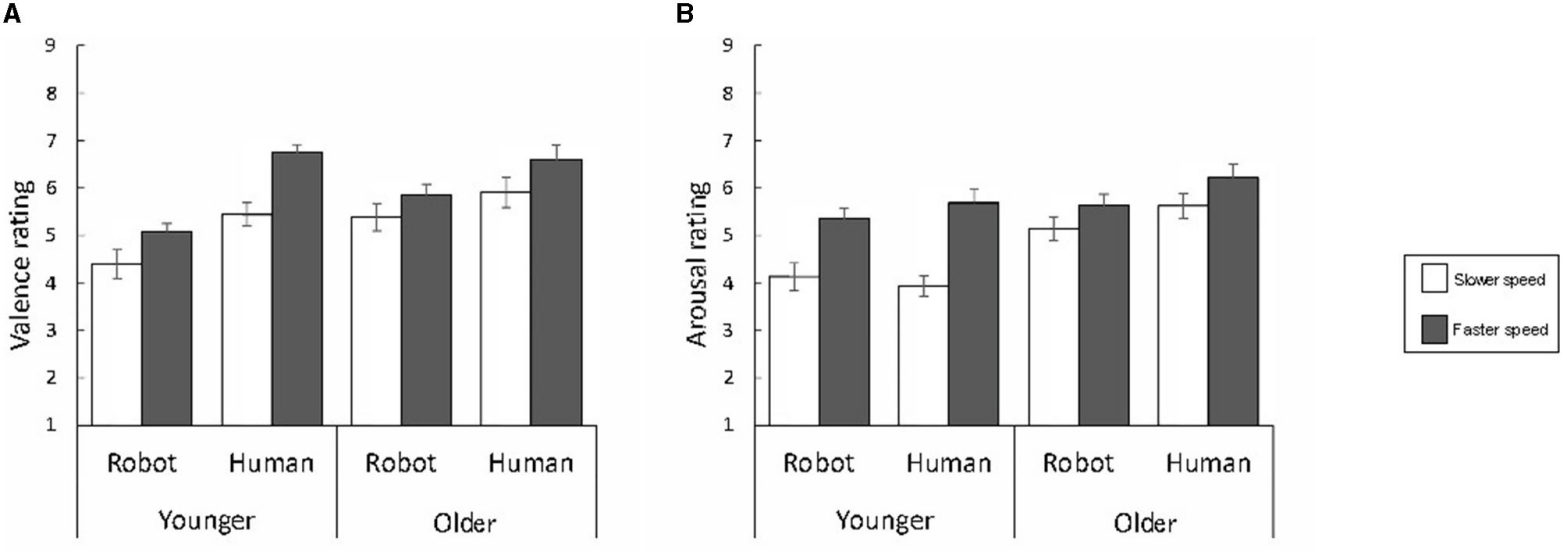
The mean and standard error of the subjective ratings for valence **(A)** and arousal **(B)**.

**Figure 3:**
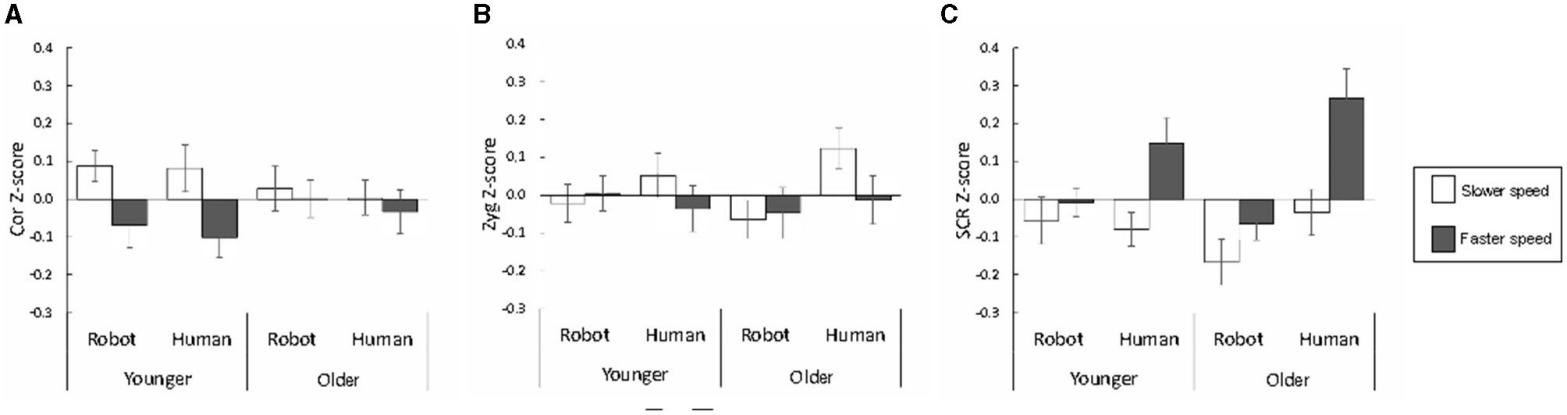
The mean and standard error of physiological signals of corrugator EMG **(A)** and zygomatic EMG **(B)**, and skin conductance response [SCR **(C)**].

Figures 2, 3 show the subjective ratings and physiological signals to the touch stimuli delivered by the two different agents at the two different speeds in the younger and older groups. We analyzed the data with three-way ANOVAs with group, speed, and agent as factors.

### 3.1 Subjective ratings

For the valence ratings, the results showed significant main effects of speed and agent, and group × agent and speed × agent interactions [*F*s_(1, 56)_ = 21.49, 47.96, 6.30, and 6.92, *p*s = 0.000, 0.000, 0.015, and 0.011, respectively]. The main effects of speed and agent revealed that touch delivered by the human agent at the faster speed induced more positive ratings. Follow-up analyses of the group × agent interaction showed that the simple main effect of the agent was significant for both the younger and older groups [*F*s_(1, 56)_ = 44.52 and 9.75, *p*s = 0.000 and 0.003, respectively], showing that the responses induced by the human agent were more positive in both groups. Follow-up analyses of the speed × agent interaction showed that the simple main effect of the speed was significant for both the robot and human agents [*F*s_(1, 112)_ = 9.11 and 28.30, *p*s = 0.003 and 0.000, respectively], indicating that both agents induced more positive experiences at the faster versus slower speed.

For the arousal ratings, the three-way ANOVA showed significant main effects of group and speed, and the group × speed and speed × agent interactions [*F*s_(1, 56)_ = 10.81, 30.33, 6.51, and 4.35, *p*s = 0.002, 0.000, 0.014, and 0.042, respectively]. The main effects of group and speed indicated that the highest arousal responses were elicited by touch delivered by the human agent to the older group at the faster speed. Follow-up analyses of the group × speed interaction revealed that the simple main effect of speed was significant for both younger and older groups [*F*s_(1, 56)_ = 32.47 and 4.37, *p*s = 0.000 and 0.041, respectively], demonstrating that both groups showed higher arousal at the faster versus slower speed. Follow-up analyses of the speed × agent interaction indicated that the simple main effect of speed was significant for both the robot and human agents [*F*s_(1, 112)_ = 18.90 and 34.67, *p*s = 0.000 and 0.000, respectively], showing that both agents elicited higher arousal at the faster versus slower speed.

### 3.2 Physiological signals

For the corrugator EMG, the ANOVA with group, speed, and agent as factors revealed that only the main effect of speed was significant [*F*_(1, 56)_ = 4.77, *p* = 0.033], showing that touch at the faster speed elicited weaker corrugator supercilii muscle activity. This suggests that positive emotions were elicited across both agents and groups.

For the zygomatic EMG, none of the main effects or interactions were significant [*F*s_(1, 56)_ < 3.93, *p*s > 0.052]. For SCR, the results revealed significant main effects of speed and agent, and significant speed × agent interaction [*F*s_(1, 56)_ = 9.21, 11.08, and 4.81, *p*s = 0.004, 0.002, and 0.032, respectively]. The main effects of speed and agent showed that touch delivered by the human agent at the faster speed elicited higher SCRs. Follow-up analyses of the speed × agent interaction revealed that the simple main effect of speed was significant for the human agent [*F*_(1, 112)_ = 14.00, *p* = 0.000], indicating that touch delivered by humans at the faster speed induced higher SCRs.

### 3.3 Attitudes toward robots

**Figure 4:**
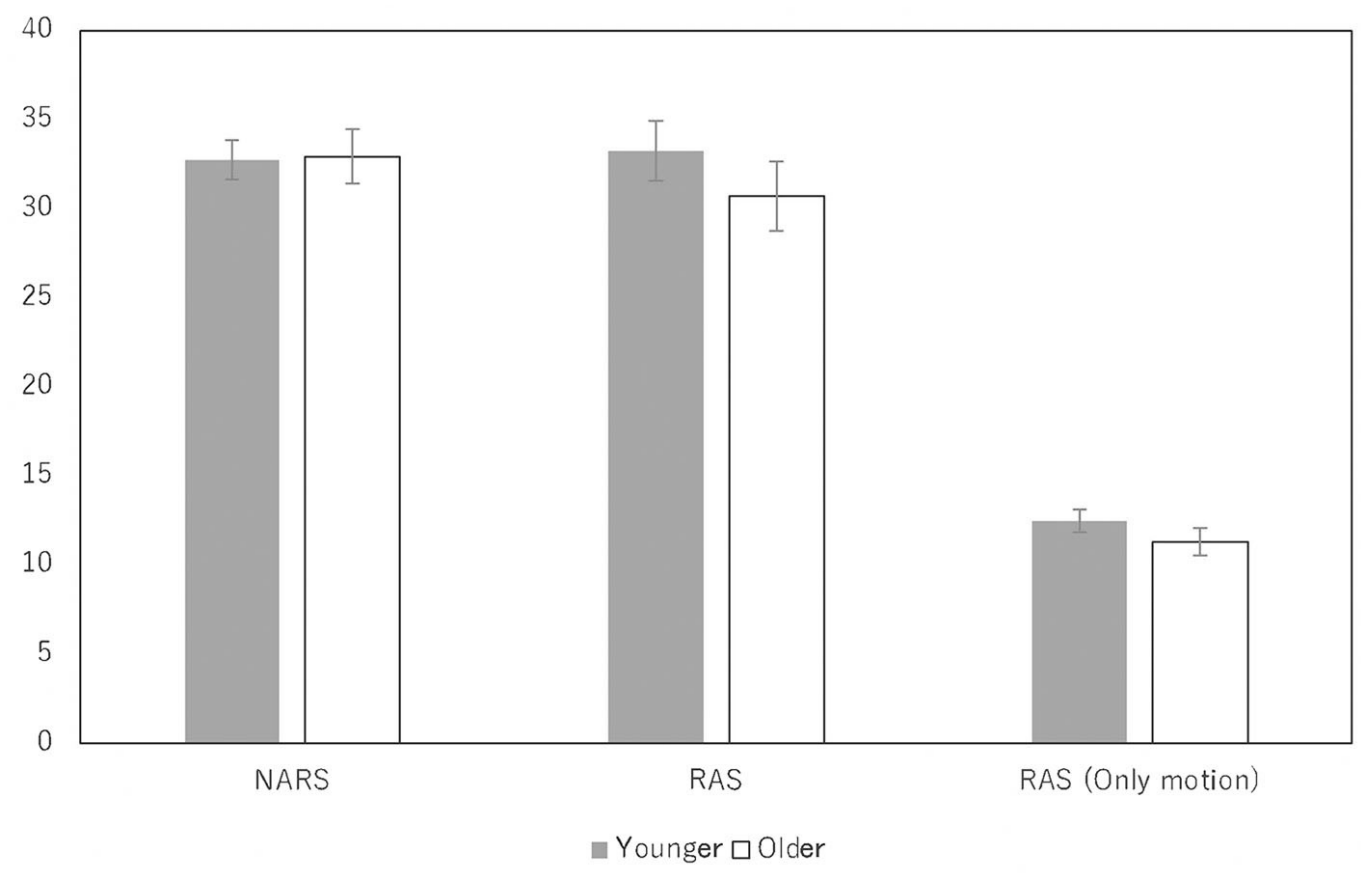
The mean and standard error of NARS and RAS, of young and old groups for robots.

Figure 4 shows the attitudes toward robots in the younger and older groups, including scores on the NARS, which evaluates robot behavior styles in human robot interaction, and scores on the RAS, which measures anxiety that prevents individuals from interacting with robots designed to assist with communication in daily life. For both scales, higher values represent a more negative evaluation. Since only robot motion (and not appearance) had a psychological influence in this study, we showed RAS scores related to motion only on the right side of Figure 4. We found no significant differences between the younger and older groups for all scales (*p*s > 0.10).

## 4 Discussion

The data regarding the subjective valence and arousal ratings and corrugator EMG activity showed that the speed modulated the emotional responses to touch strokes in the same manner across the younger and older groups. Specifically, the touch strokes at the faster (8.5 cm/s), compared with the slower (2.6 cm/s) speed induced more positive and arousing subjective responses and a greater reduction in corrugator EMG activity in both the younger and older groups. The effect of touch speed was observed for both the robot and human agents, and for both age groups. The induction of pleasant subjective and physiological emotional responses by touch strokes is consistent with several previous studies that tested touch using a brush in younger participants (e.g., Ree et al. 2019). The present results regarding the influence of touch speed on emotional responses are consistent with previous findings that touch-stimulating CT fibers at different speeds induced positive emotional responses depending on the location on the body (Ackerley et al., 2014). However, no study has examined these topics using robotic touch in older participants. To the best of our knowledge, this study is the first to provide evidence that robotic touch can induce subjective emotional and physiologically positive responses, similar to those induced by human touch, in older adults in the same way as in younger people.

Our results regarding the subjective valence and arousal ratings showed that older participants generally experienced more positive and arousing emotions than younger participants in response to touch strokes. This suggests that the emotional impact of touch-based care could be stronger in older adults, irrespective of speed or agent type.

Our results regarding the effects of touch speed across human and robot agents in older participants have a practical implication. As mentioned in the Introduction, clinical studies have shown that touch could have positive effects on physical and mental health in older people (Arnould-Taylor, 1991; Honda et al., 2016). On the other hand, the number of older adults is increasing and that of care workers is insufficient. Our results suggest that human-like therapeutic touch care could be implemented by robots to promote the health and well-being of older adults.

Several limitations of this study should be acknowledged. First, we only tested a limited number of male participants from a single country, Japan. Although we determined the sample size required to detect small effects through a *priori* power analysis, we used an alpha level of 0.05, which is potentially controversial (Benjamin et al., 2018; Lakens et al., 2018). The findings should be confirmed with a larger sample and a more conventional α level. Additionally, because attitudes toward robots may differ among cultures, the generalizability of the present findings should be confirmed in different populations. Possible gender differences should also be considered; we only tested male participants. Second, the response against the robot's stroke was inferior to the response against human stroking, as also pointed out in the previous study (Ishikura et al., 2023). However, whereas the previous study discussed potential ways to enhance the effects of the robot's stroke. The main purpose of this paper was to compare the responses of younger and older participants; notably, we used the same experimental paradigm in both studies. Finally, we used a relatively small number of subjective and physiological measures. Previous studies used additional measures such as showing that human touch reduced subjective stress and cortisol levels (Listing et al., 2010). The inclusion of such measures in future studies may enhance the overall understanding of the positive effects of robotic touch on older adults.

In conclusion, we investigated subjective and physiological emotional responses to touch strokes delivered by human and robot agents at two different speeds in younger and older participants. Subjective data from both younger and older participants showed that touch at the faster speed was more positive and emotionally arousing than that at the slower speed for both human and robot agents. Physiological data arousing than that at the slower for both human and robot agents. In short, the overall patterns of the older group's responses were similar to those of the younger group. These results suggest that robot-delivered stroke touch can induce positive emotional responses in older adults.

## Data availability statement

The original contributions presented in the study are included in the article/supplementary material, further inquiries can be directed to the corresponding author.

## Ethics statement

The studies involving humans were approved by Nara Institute of Science and Technology. The studies were conducted in accordance with the local legislation and institutional requirements. The participants provided their written informed consent to participate in this study.

## Author contributions

TI: Data curation, Investigation, Writing – review & editing. WS: Conceptualization, Formal analysis, Funding acquisition, Methodology, Writing – original draft. JT: Conceptualization, Data curation, Funding acquisition, Methodology, Project administration, Writing – original draft. AY: Supervision, Writing – original draft. S-GC: Supervision, Writing – review & editing. MD: Supervision, Writing – review & editing. SY: Supervision, Writing – review & editing. TO: Funding acquisition, Supervision, Writing – review & editing.

## Footnotes

^1^ https://www.universal-robots.com/e-series

^2^HITOHADA GEL, EXSEAL Co., Ltd, Japan. http://www.exseal.co.jp/english/creative/hitohada.html

^3^The previous study (Ishikura et al., 2023) also collected NARS and RAS from the younger participants, but did not analyze the data.
